# Inclination not force is sensed by plants during shoot gravitropism

**DOI:** 10.1038/srep35431

**Published:** 2016-10-14

**Authors:** Hugo Chauvet, Olivier Pouliquen, Yoël Forterre, Valérie Legué, Bruno Moulia

**Affiliations:** 1 Aix-Marseille Univ., CNRS, IUSTI UMR 7343, 13453 Marseille Cedex 13, France; 2 Integrative Physics and Physiology of Trees (PIAF), INRA, Univ. Clermont-Auvergne, 63000 Clermont-Ferrand, France

## Abstract

Gravity perception plays a key role in how plants develop and adapt to environmental changes. However, more than a century after the pioneering work of Darwin, little is known on the sensing mechanism. Using a centrifugal device combined with growth kinematics imaging, we show that shoot gravitropic responses to steady levels of gravity in four representative angiosperm species is independent of gravity intensity. All gravitropic responses tested are dependent only on the angle of inclination from the direction of gravity. We thus demonstrate that shoot gravitropism is stimulated by sensing inclination not gravitational force or acceleration as previously believed. This contrasts with the otolith system in the internal ear of vertebrates and explains the robustness of the control of growth direction by plants despite perturbations like wind shaking. Our results will help retarget the search for the molecular mechanism linking shifting statoliths to signal transduction.

The ability of living organisms to feel gravity is a key function for their development and adaptation. In vertebrates, the gravity sensor (otolith organ) consists of a mass of calcium carbonate bodies within a gelatinous matrix attached to hair cells^1^. When acceleration or inclination occurs the mass moves and deforms the hair cells. Otolith organs are therefore sensitive to both the direction and amplitude of the gravity vector. As a consequence, they do not discriminate between a linear acceleration and an inclination, which has important physiological implications^2^. In plants like in animals, gravisensing relies on small bodies called statoliths. But these bodies are starch-filled organelles that occurs inside specialized cells, the statocytes. Whether this system allows the plants to discriminate between linear acceleration (e.g. induced by wind shaking) and inclination such as stem lodging remains unknown.

When the plant is inclined, the statoliths sediments in the direction of the gravity inside the statocyte^3^. A redistribution of auxin transporters is then triggered in the statocyte^4^. According to the “Cholodny-Went” hypothesis, the resulting auxin gradient causes asymmetric growth and global bending of the organ, a response called gravitropism^4^,^5^,^6^. However the exact sensing mechanism is still a topic of very active research^7^. Two different hypotheses have been proposed to explain how the statoliths are detected. Some consider that the transduction is triggered by the proximity of statoliths to subcellular elements^8^,^9^, when others suggest that the statocytes detect the pressure exerted by the statoliths on cell elements^10^ (statolith pressure hypothesis). Although the key role played by the statoliths in the gravity perception is generally accepted, the reduced gravitropic response of starchless *arabidopsis* mutants deprived of statolith sedimentation tends to prove that another more ancient and parallel sensing mechanism may be at work^11^,^12^. This ancient mechanism has been hypothesized to work similarly to the statolith pressure hypothesis except that the pressure is due to the weight of the overall protoplast (protoplast pressure hypothesis). These scenari have different implications for the plant gravitropism. Assuming that the statocytes detect the weight of the statoliths and/or of the protoplast means that they act as force sensors and implies that gravitropism should be sensitive to the gravity intensity and any linear acceleration. This should not be the case if statocytes detect the position of the statoliths. Studying the role of gravity intensity on the response of plants to inclination is then a way to discriminate between the hypotheses.

So far, the influence of gravity intensity on plant gravitropism has been mainly studied for transient stimuli using centrifuges in microgravity conditions in space^13^ or clinostats (a device that rotate plants in the horizontal plane to compensate for the sensing of Earth gravity *g*^14^). It was found that the response was proportional to the dose of the transient stimulus *g*_*eff*_ × *t*, where *g*_*eff*_ is the effective gravity and *t* the exposure time^15^,^16^. Paradoxally, the response of plants to a permanent gravity stimulus^12^,^14^,^17^, the environment in which plant species have evolved, has been much less studied. Under constant *g* on Earth^14^,^17^, it was shown that the gravitropic response of a plant inclined at an angle *θ*_*init*_ relative to the vertical is proportional to the sine of the angle of inclination^18^,^19^,^20^,^21^,^22^. The text-book explanation of this sine law^19^ is that the statocytes in the responding organ sense the force exerted by the weight of the statoliths on the side of the cell, the magnitude of which varies as the sine of the inclination angle. As the weight is also proportional to gravity intensity (see Fig. 1), we should then expect a gravitropic response proportional to *g* sin(θ_init_)^12^. Therefore both types of studies based on transient and permanent stimulus may seem to favor a force-sensing mechanism. However the two approaches (varying the intensity of the effective gravity and the inclination to the gravity vector in a permanent way) have never being combined so that the consistency of the proposed sensing mechanism has never been assessed.

## Results

To understand how cellular gravity sensing works we investigated the combined effects of permanent inclination and gravity intensity on the response of different plant species and shoot types. We chose to study a broad range of effective gravity from 0.1*g* to 3 *g*. To put this in context, wind and rain on Earth can induce transient accelerations of up to 3 *g* in aerial organs^23^, while gravity on the Moon is just 0.17 *g*^24^.

### A proper dimensionless measure for the gravitropic response

A crucial first step is to define a suitable measure of the gravitropic response^12^. Several responses have been used in the past to measure gravitropism such as the variation in tip angle over time^20^,^25^ or the more detailed distribution of the curvature rate and of the differential growth between the upper and lower side of the organ^17^. Recently, biomechanical models of plant gravitropism have emphasized the need to measure the full spatio-temporal shaping of the plant and its elongation growth to properly measure gravisensing^12^,^26^. The shape can then be quantified using curvature *C* as defined in differential geometry (i.e. the rate of change in angle *θ* with spatial position *s* along the organ *dθ*/*ds*) and it is possible to relate curvature changes along the organ with the changes in the tip angle.

A time-lapse sequence of such a spatio-temporal response is shown in Fig. 2a for a *Triticum aestivum* wheat coleoptile grown in the dark under *g* and initially inclined at *θ*_*init*_ = 50°. Using image processing techniques (Supplementary Fig. S1b), we extracted the length *L*(*t*) (Fig. 2b), the tip angle *θ*_*tip*_(*t*) (Fig. 2c) and the base angle *θ*_*base*_(*t*) of the coleoptile as functions of time. The coleoptile started to bend during a transient phase of about 20 min, then over a longer period the coleoptile tip angle *θ*_*tip*_ increased linearly with time (the velocity *dθ*_*tip*_/*dt*, is constant, see shaded area in Fig. 2c), until a vertical position *θ*_*tip*_ = 0 was reached after several oscillations (Fig. 2c). During this whole process, *θ*_*base*_ remained constant (not shown) and the plant grew at a constant elongation rate *dL*/*dt* (Fig. 2b). For the following experiments, we focused on the initial stage of the dynamics when the plant curvature was small and proprioception (the tendency of plants to sense their own curvature independently of any other stimulus) was negligible compared to gravisensing^17^,^26^.

So far, the most accurate quantitative monitoring of the local reponse to gravisensing at the initial stage, has usually been based on the difference in the relative elongation growth rates 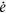 between the two sides of each segment of the shoot^12^,^27^,^28^ 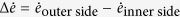. 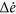 has been shown to be directly related to the rate of change in curvature *dC*/*dt* by the following relation: 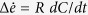, where *R* is the radius of the shoot^26^. However 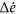 is still confounding the results of two different biological processes (i) the overall amount of auxin in the segment due to the longitudinal polar auxin transport which controls the mean relative growth rate of the segment 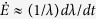, where *λ* is the length of the segment and (ii) the transverse redistribution of the auxin available at that time in the segment by lateral transport. This lateral transport locally modifies the amount of auxin distributed to the two sides of the shoot and induces a relative differential growth 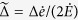. Only this relative differential growth 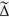 is triggered by the response to gravisensing^4^ (see Bastien *et al*.^26^ for mathematical details). Therefore a measure of the direct response to gravisensing is 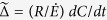. Beside focusing on the consequence of the changes in the lateral transport of auxin, 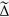 has also the advantage to be a dimensionless quantity; it does not depend on the arbitrary choice of units. Following Bastien *et al*.^26^, the mean relative growth rate 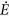 was homogeneised over the whole growth zone 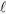 and defined as 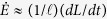. In our experiments the plant organs were initially straight so we could also approximate curvature as: 
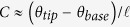
. The dimensionless gravitropic response 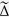 is then given by


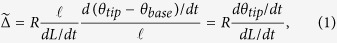


which can be computed from the curves in Fig. 2b,c by measuring *dθ*_*tip*_/*dt* and *dL*/*dt* in the respective shaded regions. 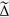 provides a measure of the gravitropic response corrected from the organ size *R* and the effects of the growth rate *dL*/*dt*, that can change the rate of curvature independently of any gravitropic stimuli. Note that its measure does not require to estimate the size of the growth zone 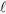 since the change of curvature and the mean relative growth rate are estimated over the same size 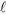. 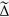 is then not affected by any changes of 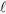 during the experiments as long as the zone of curvature coincide with the zone of elongation.

To test the validity of this approach we grew wheat coleoptiles at different temperatures (17–32 °C) in order to vary their growth rates (Fig. 2d). For a given initial inclination *θ*_*init*_, decreasing the growth rate decreased the rate of change of the tip angle. However, *dθ*_*tip*_/*dt* remained proportional to *dL*/*dt* as shown for two different ranges of angles of initial inclination Fig. 2d, showing that 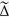 only depends on *θ*_*init*_ and is a relevant measure of the gravitropic response.

### The gravitropic response obeys a sine-law independent of gravity intensity

We next measured such gravitropic responses under *g* varying the initial inclination of coleoptiles from 0° (a vertical coleoptile pointing upwards) to 180° (a vertical coleoptile pointing downwards). The gravitropic response increased almost linearly from zero for *θ*_*init*_ = 0, to reach a maximum of approx. 0.6 around *θ*_*init*_ = 90° and decreased back to zero for higher inclination angles (Fig. 3a, circles). A sine function fits the data well confirming the robustness of the sine law for a gravisensing response over the whole range of inclinations^12^.

We designed a setup to experimentally alter the magnitude of *g*_*eff*_ while imaging the spatio-temporal response of the organs at sufficient resolution. Plants were grown in a chamber placed in a device that rotates at an angular velocity Ω about a vertical axis (Fig. 3b). A plant at a position *r* from the center of rotation is thus subject to a centrifugal acceleration *r*Ω^2^, resulting in 
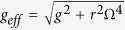
 inclined from the vertical at an angle arctan(*r*Ω^2^/*g*). Using this setup, it is thus possible to independently study the influence of *θ*_*init*_ and *g*_*eff*_ on the gravitropic response, at least when *g*_*eff*_ > *g*. Note that both the initial inclination *θ*_*init*_ of the plant and the plant tip angle *θ*_*tip*_ were measured relative to the local *g*_*eff*_ (Fig. 3b). Before quantifying the gravitropic response in this set-up, it was necessary to check that the mean relative growth rate 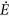 was not affected by the intensity of gravity, as thigmomorphogenetic effect induced by hypergravity have been reported^29^. For the range of gravity intensity used in this study, the growth rate *dL*/*dt* and the size of the growth zone *l* do not depend on *g* (Supplementary Figs S2 and S4), so that the gravitropic response could be quantified using (1).

**Figure 1:**
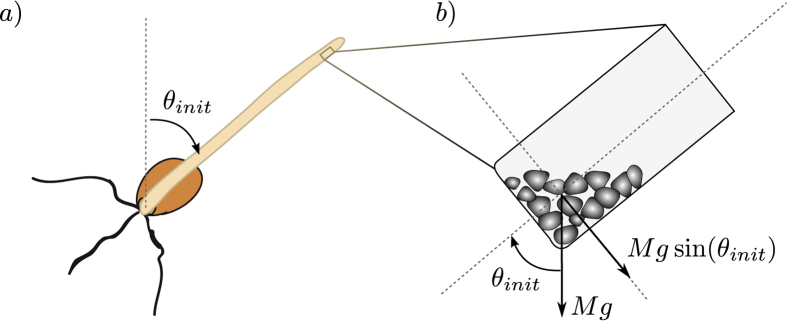
Schematic representation of the g sine law for gravisensing of lateral forces. (**a**) Wheat coleoptile inclined at an initial angle *θ*_*init*_ from the direction of gravity. (**b**) Close-up view of statocyte containing sedimented statoliths. *Mg* sin(*θ*_*init*_) is the force induced by the buoyancy-corrected mass *M* of statoliths grains on the lateral cell membranes.

**Figure 2:**
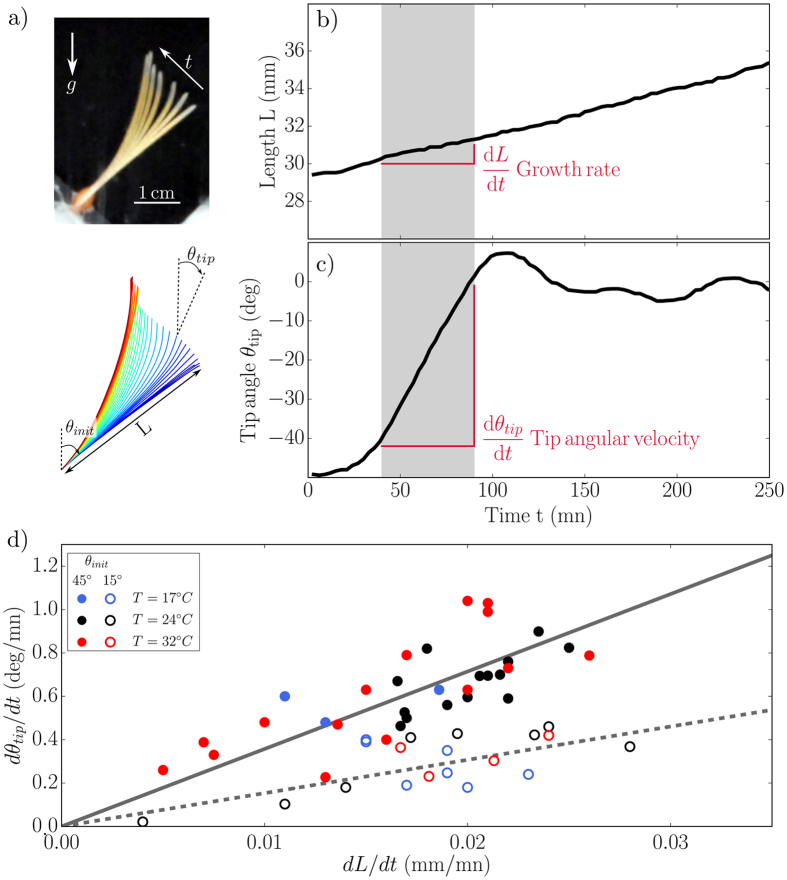
Example of an intrinsic gravitropic response of a plant shoot. (**a**) Time-lapse image of growth response of a wheat coleoptile initially inclined at *θ*_*init*_ = 50° from the direction of gravity (top panel) and corresponding topological skeleton extracted by image processing (bottom panel, colours correspond to different time points). (**b**) Change in the total length L of the coleoptile shown in (**a)** over time *t*. (**c**) Changes in the tip angle *θ*_*tip*_ of the coleoptiles shown in (**a)** over time *t*. Shaded areas in (**b**,**c**) indicate the time-range for the linear fit used to estimate *dθ*_*tip*_/*dt* and *dL*/*dt*. (**d**) Tip angular velocity *dθ*_*tip*_/*dt* plotted as a function of growth rate *dL*/*dt* for plants grown at different temperature from 17 °C to 32 °C. (•) 40° < *θ*_*init*_ < 50°; (○) 10° < *θ*_*init*_ < 20°.

**Figure 3:**
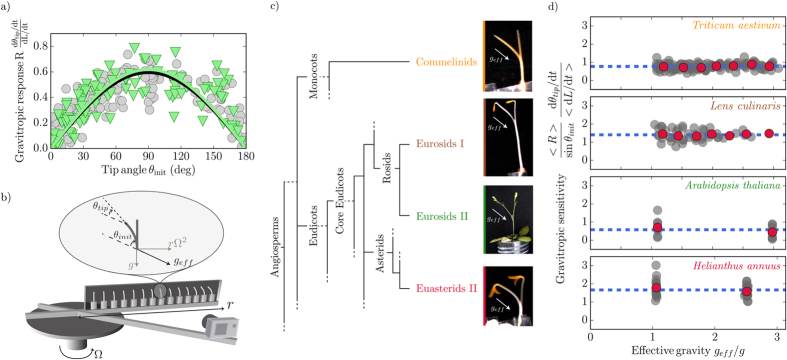
Gravitropic response is independent of effective gravity. (**a**) Normalized gravitropic response of wheat coleoptiles as a function of inclination angle *θ*_*init*_ at 1*g* (grey circles) and 2.5 *g* (green triangles). Black and green curves are the respective fitted sine functions. (**b**) Sketch of the experimental setup. Plants in a chamber (60 cm × 16 cm × 3 cm) are rotated at an angular velocity Ω about a vertical axis. Centrifugation creates an effective gravity *g*_*eff*_. *θ*_*init*_ and *θ*_*tip*_ are the initial angle of the plant and the angle of the tip relative to the direction of *g*_*eff*_. (**c**) Simplified phylogenetic tree (adapted from the Angiosperm Group Phylogeny III classification, http://www.mobot.org/mobot/research/apweb/) showing relationships between the four species studied (from top to bottom): *Triticum aestivum*, wheat coleoptile; *Lens culinaris*, lentil stem; *Arabidopsis thaliana*, Arabidopsis inflorescence stem; *Helianthus annuus*, sunflower hypocotyl. (**d**) Gravitropic sensitivity 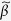 plotted as a function of the gravity intensity for the four species in (**c**). As the elongation rate was independent of *g*_*eff*_ (Supplementary Fig. S2), a mean elongation rate was used to compute 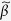. Means of values in bins of equal ranges in gravity intensity are shown (red circles) with the mean of the whole dataset (dashed blue lines).

Figure 3a (triangles) gives the response to inclination of wheat coleoptiles when the intensity of the effective gravity is more than twice the Earth gravity (*g*_*eff*_ = 2.5 *g*). Unexpectedly, the response is the same at 2.5 *g* as at *g*. The two fits by a sine function coincide, suggesting that gravity intensity itself plays no role in the gravitropic response. This result cannot just be due to saturation of the gravisensing of the lateral force *Mg* sin(*θ*_*init*_) exerted by the statoliths on the cell at high gravity levels (Fig. 1). Indeed, when this lateral force is reduced by lowering the inclination angle (i.e. when sin(*θ*_*init*_) becomes small), the response is still the same at *g* as at 2.5 *g*, and no gravisensing threshold nor plateau could be observed.

This result suggests that the gravitropic response can be described by a sine law 
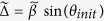
 but not by the “*g* sine law” predicted for a gravitational force sensor. 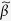 can thus be considered to be the intrinsic sensitivity of the sensor to angular inclination from the gravity direction^26^. To further assess that beta tilde is indeed independent of the gravity intensity, we extended the range of effective gravity investigated (1 < *g*_*eff*_/*g* < 3) and we plotted the gravitropic sensitivity 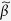 estimated as


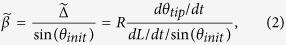


as a function of the *g*_*eff*_ normalized to *g* (Fig. 3d). The data clearly rule out the linear relationship between the gravitropic sensitivity and *g*_*eff*_ predicted by the “*g* sine law” (P-value < 10^−3^, see Supplementary Table S1). Moreover, the sensitivity 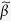 was found to be independent of gravity in the range investigated (the slope of the linear fit 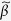 as a function of (*g*_*eff*_/*g*) is negligible at 0.05 ± 0.03 ≪ 1, see Methods).

We carried out similar experiments to those conduted in wheat with *Lens culinaris* (lentil) vegetative stems, *Helianthus annuus* (sunflower) hypocotyls and *Arabidopsis thaliana* inflorescence stems. The four species studied are respectively from the most populated clades of land angiosperms: the commelinid monocots and the eurosid I and II and eu-asterid II dicots (Fig. 3c). The sensitivity 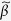 of each of the four species was found to be independent of gravity (Fig. 3d). Interestingly, gravitropic sensitivity 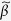 differed significantly between species (Supplementary Fig. S3).

### Gravity-independent sensing extends to hypogravity conditions

**Figure 4:**
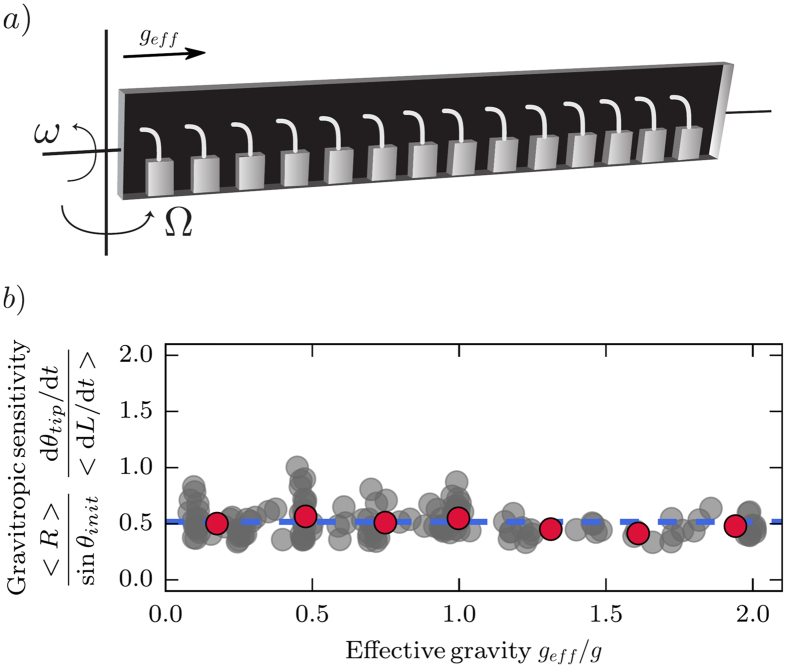
Clinostat-centrifuge experiments showing gravity-independent gravitropic sensitivity under both hypo and hypergravity. (**a**) Sketch of the clinostat mounted on the rotating table, where *ω* is the angular velocity around the horizontal axis and Ω is the angular velocity of the centrifugal motion. (**b**) Gravitropic sensitivity of wheat coleoptiles as a function of the effective gravity (initial inclination *θ*_*init*_ = 90°). Means of values in bins of equal ranges in gravity intensity are shown (red circles) with the mean over the whole dataset (dashed blue line).

To investigate whether the insensitivity to gravity-intensity extends to hypogravity conditions (*g*_*eff*_ < *g*), we modified the setup by adding to the centrifuge rotating table a clinostat to rotate the plant chamber around its horizontal axis at an angular velocity *ω* = 4 rotations per min. The clinostated growth chamber was then rapidly rotated around the vertical axis at an angular velocity Ω on the rotating table as before (Fig. 4a). Clinostats have been shown to compensate the gravisensing in the plane perpendicularly to the rotation axis, while creating a negligible centrifugal force^15^. In this configuration, plants were only sensing an horizontal *g*_*eff*_. Experiments were performed on wheat coleoptiles in the range 0.1 *g* *<* *g*_*eff*_ < 2 *g*. The gravitropic response was again found to be independent of the magnitude of *g*_*eff*_ over the whole range (Fig. 4b). The absolute value of 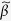 was however less than in the experiments without the clinostat which is possibly due to the reaction of the plants to repeated mechanical bending during clinorotation^12^.

## Discussion

We conclude that the gravitropic response of shoots is independent of gravity intensity. Unlike animals, plants can sense inclination irrespective of gravitational or inertial acceleration intensity to control their posture. This implies that statocytes do not measure the force exerted by the weight of the statoliths or by the protoplast on an area of the cell membrane or wall. Statocytes should therefore be considered as position sensors not force sensors. This is consistent with statolith displacement being required for gravisensing^9^, but apparently contradicts the “dose response” measured for transient stimuli^15^,^16^ where the response increased with both the exposure time and gravity intensity. Possibly for transient stimuli the time of statolith sedimentation, which depends on *g*, become larger than the exposure time. The transient “dose response” would then reflect the *g*-dependent physics of the sedimentation rather than the sensing mechanism. The results also challenge our knowledge of the physics of assemblies of frictional grains^30^,^31^,^32^. The observation of no angular threshold on the gravitropic response (sine law, Fig. 3a) means that the statoliths move even at small inclination unlike granular packing in which jamming happens^32^, suggesting that Brownian^30^ or active fluctuations^31^ play a key unlocking role for the statolith motion. Our result also constrains the different molecular hypotheses of gravitropic perception. It gives quantitative support to mechanisms involving the proximity of the statoliths to subcellular elements (endoplasmic reticulum^8^, actin cytoskeleton^9^) or a change of the intracellular trafficking due to statolith asymmetric distribution within the cell. But, it dismisses mechanisms based on the detection of pressure due to the weight of statoliths (statolith pressure hypothesis^10^) or of the whole protoplast (protoplast pressure hypothesis^11^,^12^). Finally, this result involves that any molecular models regulated by the intensity of gravity are unlikely to explain the gravitropic response observed at the plant scale. Our method can now be used to analyse other organs, like roots, and to phenotype mutants (including the reduced starch mutants) to provide direct insights into the molecular mechanisms^8^,^9^,^10^ involved in the positional sensing of statoliths in statocytes.

## Methods

### Plant materials and growth condition

#### Wheat coleoptiles

Wild-type wheat seeds (*Triticum aestivum* cv Demeter) were used for all experiments on wheat coleoptiles. Seeds were initially glued with floral glue at the top of small plastic boxes filled with cotton wool, one seed per box (see Supplementary Fig. S1a). The seeds were glued with the germ pointing downward toward the cotton wool in order to ensure that the coleoptiles initially grew as straight as possible. The boxes were put in a humidity chamber where a spray of tap water was injected 5 times during 15 min daily (EasyGreen^®^ sprouter, automatic germination system, http://www.easygreen.com/). Seeds were grown in the dark at 24 °C. Experiments were carried out when the coleoptiles were between 1.5 and 2.5 cm tall (about 4 days after germination), before the leaves emerged.

#### Lentil stems

Wild-type lentil seeds (*Lens culinaris* cv Germline) were grown in the same conditions as the wheat. Seeds were glued on their flat surface at the top of the box with the germ facing the cotton as sketched in Supplementary Fig. S1c. Experiments were carried out when the shoots were between 2 and 3.5 cm tall typically 4 days after germination.

#### Sunflower hypocotyls

Wild-type sunflower seeds (*Helianthus annuus* cv Germline) were grown in soil in individual cylindrical containers. They were grown in the dark at 24 °C. Experiments were carried out when the hypocotyls were between 2 and 4 cm tall, typically 4 days after germination.

#### Arabidopsis inflorescences

*Arabidopsis thaliana* Columbia-0 seeds were grown in soil in individual pots in a growth chamber controlled for light, humidity and temperature. Experiments were carried out when inflorescences were about 4 to 5 cm tall typically after 3–4 weeks. Before putting the plants on the rotating table for experiments, all leaves and lateral shoots were removed.

### Rotating experimental setup

We built a novel setup based on the clinostat-centrifuge^15^,^22^ that allowed time-lapse imaging of plant kinematics (Fig. 3b). This setup consists of a closed chamber (60 cm long, 16 cm high, 3 cm wide) attached to a rotating table (Shimpo RK-3E, Shimpo Kyoto Japan) with an accurate control of the rotation angular velocity Ω between 0.4 and 1 rotation per second. A camera with a wide-angle lens was fixed to the table and set to take pictures of the whole box every 3 min. The camera was synchronized with a flash filtered in the green safelight waveband when necessary to avoid phototropism. The whole setup was placed in the dark room at 24 °C as standard or at other temperatures as indicated. Individual plants were initially grown vertically under Earth’s gravity. At the start of the experiments plants were gently positioned in the chamber at different radial positions *r* from the center of rotation and at a different initial inclination *θ*_*init*_. Rotation was then switched on. When the clinostat was also used, the whole chamber was rotated along its horizontal axis by a motor mounted on the rotating table at an angular velocity *ω* equal to 4 rotations per min (Fig. 4a). Then the rotating table was turned on.

### Image analysis

To extract the topological skeleton of plants from photographs, specific software has been developed. The principle is shown in Supplementary Fig. S1b. A perpendicular line is first drawn through the base of the plant. Extrapolating along the line, the two outer edges of the plant organ are found from the maximum gradients of the greyscale profile. The first point of the skeleton is defined as the midpoint between the two outer edges and the diameter is defined as the distance between the edges. The line was then transposed along the shoot to estimate the next skeleton point. The typical distance between two successive lines was 0.3 pixel, which gave a good resolution. The orientation of the line varied along the shoot to remain perpendicular to the shoot inclination estimated from the skeleton points already extracted. For wheat, the process was complete when the diameter of the plant fell below a critical value. For the other plants when leaves or cotyledons were present at the tip, at the start of an experiment a line was drawn with black ink just below the leaves to serve as an end-point for image processing. From the coordinates of the skeleton, one can compute the length *L* of the plant, and the local inclination as a function of the curvilinear abscissa. The tip angle *θ*_*tip*_ was defined as the average inclination at the tip over a length equal to two mean diameters.

### Statistics

In Fig. 3c the hypothesis of a linear dependence of the gravitropic response as a function of *g* sin *θ* was tested using a linear regression model, *ax* + *b*, with the ordinary least squares method (Python StatsModels module version 0.6.1). The results are given in Supplementary Table S1. To test the *g* sine law, the probability that the observed intercept *b* could result from sampling from a population following the *g* sine law (i.e. the null hypothesis, the intercept is equal to zero) was calculated using the Student’statistics. If the resulting P-values are low, then the null hypothesis of the *g* sine law can be rejected. The Supplementary Fig. S3a shows a comparison of gravitropic sensitivity between the four species used in this study. From an analysis of variance we can reject the null hypothesis that all these species have the same gravitropic sensitivity (P-value = 4.02 × 10^−54^, obtained from an F-test). In addition comparison between pairs of species (using the Tukey HSD test) shows that gravitropic sensitivities differ significantly from each other. Results are given in Supplementary Fig. S3b.

## Additional Information

**How to cite this article**: Chauvet, H. *et al*. Inclination not force is sensed by plants during shoot gravitropism. *Sci. Rep.* **6**, 35431; doi: 10.1038/srep35431 (2016).

## Supplementary Material

Supplementary Information
