# Supplementary material for: Inclination not force is sensed by plants during shoot gravitropism
Source: Sci Rep. 2016 Oct 14;6:35431. doi: 10.1038/srep35431 (PMC5064399; doi:10.1038/srep35431)
Supplement: Supplementary Information [file srep35431-s1.pdf]

Inclination not force is sensed by plants during shoot gravitropism

Hugo Chauvet[1,2], Olivier Pouliquen[1,*], Yoël Forterre[1], Valérie Legué[2], and Bruno Moulia[2]

[1]Aix-Marseille University, CNRS, IUSTI UMR 7343, 13453 Marseille Cedex 13, France.
[2]Integrative Physics and Physiology of Trees (PIAF), INRA, Univ. Clermont-Auvergne, 63000 Clermont-Ferrand, France.
[*]olivier.pouliquen@univ-amu.fr

ABSTRACT

Gravity perception plays a key role in how plants develop and adapt to environmental changes. However, more than a century after the pioneering work of Darwin, little is known on the sensing mechanism. Using a centrifugal device combined with growth kinematics imaging, we show that shoot gravitropic responses to steady levels of gravity in four representative angiosperm species is independent of gravity intensity. All gravitropic responses tested are dependent only on the angle of inclination from the direction of gravity. We thus demonstrate that shoot gravitropism is stimulated by sensing inclination not gravitational force or acceleration as previously believed. This contrasts with the otolith system in the internal ear of vertebrates and explains the robustness of the control of growth direction by plants despite perturbations like wind shaking. Our results will help retarget the search for the molecular mechanism linking shifting statoliths to signal transduction.

Additional information

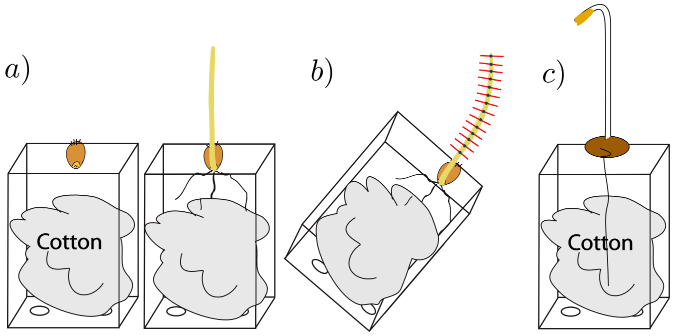

Fig. S1: Plant growth and analysis. a, Method used for growing wheat seeds in individual boxes (35 mm × 25 mm × 10 mm) containing moistened cotton wool. Note the germ of the grain is pointing downward. **b**, Sketch of the image processing technique used to extract the skeleton from each time-lapse image. Greyscale profiles are extracted along the red lines to find the edges and center of the shoot. The extraction are done from the bottom to the top. The red lines are perpendicular to the local shoot orientation. **c**, Method for growing lentil seeds. Note the seed is flat against side of the box.

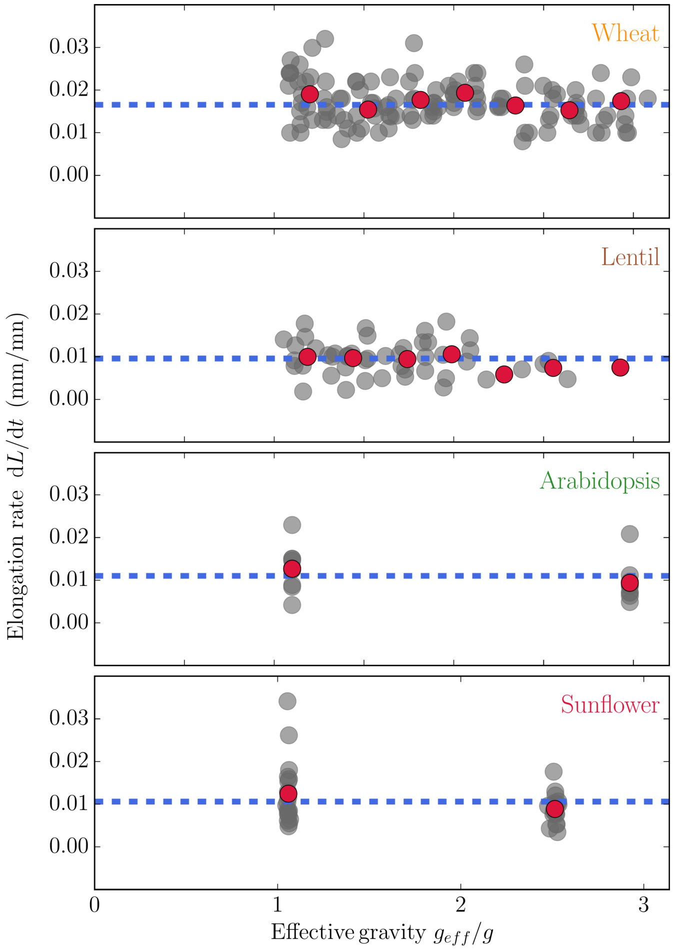

Fig. S2: Elongation rate dL/dt **in mm/min plotted as a function of effective gravity** g_{eff}/g for wheat coleoptiles, lentil stems, Arabidopsis inflorescence stems and sunflower hypocotyls. Red circles indicate averages of values in bins of equal ranges in gravity intensity, and dashed blue lines the means over the whole dataset.

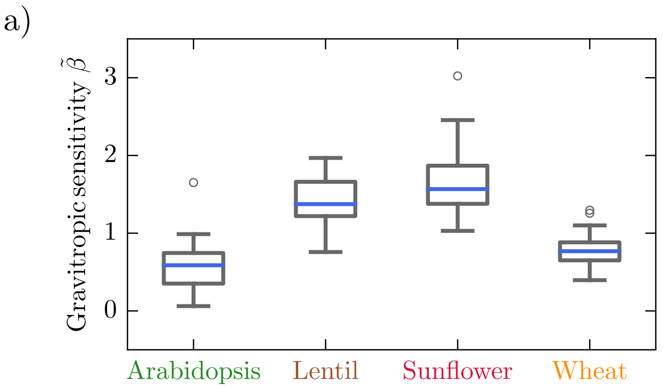

b)

	Wheat	Lentil	Arabidopsis
Lentil	3.6×10^{-14}	–	–
Arabidopsis	2.1×10^{-2}	4.7×10^{-14}	–
Sunflower	3.6×10^{-14}	8.4×10^{-5}	3.6×10^{-14}

Fig. S3: Comparison of gravitropic sensitivity in different plant species. **a,** Boxplot of the gravitropic sensitivity $\tilde{\beta}$ of Arabidopsis inflorescence stems, lentil stems, sunflower hypocotyls, and wheat colecoptiles. Blue lines represent the median for each dataset; open circles represent outliers. **b,** P-values obtained from a Tukey HSD test comparing mean gravitropic sensitivities between each species. Computation was done with Python StatsModels module version 0.6.1.

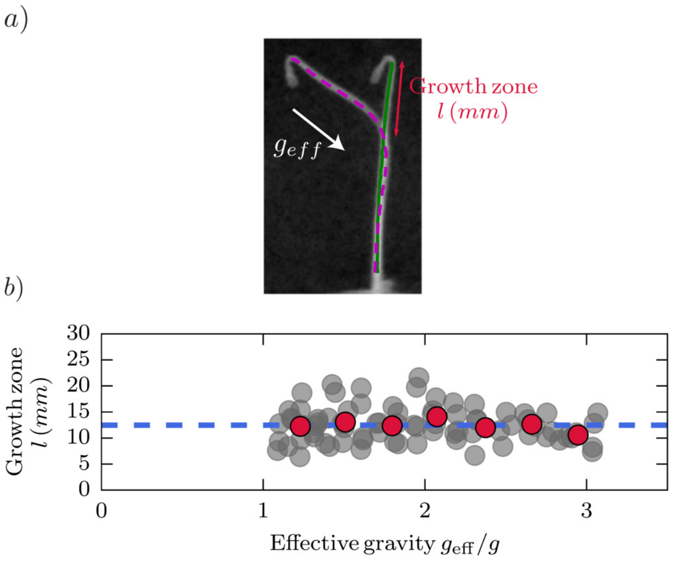

Fig. S4: Size of the growth zone l in mm plotted as a function of the effective gravity g_{eff}/g for lentil stems. **a,** Superposition of the initial and final images. The growth zone is estimated from the first part of the stem that bend to the apex of the shoot (red arrow). **b,** Evolution of the size of the growth zone for all the lentil stems of our experiments as a function of the effective gravity intensity. Red circles indicate averages of values in bins of equal ranges in gravity intensity, and dashed blue lines the means over the whole dataset.

	Wheat	Lentil	Arabidopsis	Sunflower	Wheat on clinostat-centrifuge
Sample size	119	48	20	38	144
R^2	0.038	0.003	0.043	0.043	0.026
slope a	0.055 ± 0.025	-0.037 ± 0.096	-0.130 ± 0.081	-0.113 ± 0.086	-0.038 ± 0.020
intercept b	0.663 ± 0.051	1.467 ± 0.169	0.839 ± 0.182	1.872 ± 0.174	0.548 ± 0.019
P-Value for b	3.04×10^{-24}	3.14×10^{-11}	3.26×10^{-4}	9.17×10^{-13}	7.30×10^{-60}

Table S1: Linear fit of gravitropic sensitivity with effective gravity in the four plant species. Ordinary least squares regression, $ax+b$, on the gravitropic sensitivity of wheat coleoptiles, lentil stems, Arabidopsis inflorescence stems, and sunflower hypocotyls. R^2 is the squared Pearson coefficient. The slope a and intercept b values are given $\pm$ the standard error. P-values obtained from Student's t-distribution are given for the intercept. Computation was done with Python StatsModels module version 0.6.1.
